# Risky Sexual Behavior and Associated Factors among Adolescents Aged 15-19 Years at Governmental High Schools in Aksum Town, Tigray, Ethiopia, 2019: An Institution-Based, Cross-Sectional Study

**DOI:** 10.1155/2020/3719845

**Published:** 2020-08-21

**Authors:** Mengesha Srahbzu, Enguday Tirfeneh

**Affiliations:** ^1^Department of Psychiatry, College of Medicine and Health Science, University of Gondar, Ethiopia; ^2^Department of Psychiatry College of Health Sciences and Comprehensive Specialized Hospital, Aksum University, Ethiopia

## Abstract

**Introduction:**

The impact of risky sexual practice on the general health of adolescents is enormous; little attention has been given on identification and intervention plans. Therefore, the aim of this study was to find the magnitude of risky sexual behavior and associated factors among adolescents aged 15-19 years in high schools at Aksum town, Tigray, Ethiopia.

**Methods:**

An institution-based cross-sectional study was conducted at governmental high schools of Aksum town. We recruited a total of 659 adolescents aged 15-19 years by using a systematic random sampling technique. Data was collected with a face-to-face interview. An Amharic version of the sexual risk behavior scale was used to measure risky sexual behaviors. The patient health questionnaire 9, the Oslo-3 social support scale, and an adverse childhood experience questionnaire were used to assess the factors. The coded data were entered into EpiData v.4.1 and analyzed using Statistical Package for the Social Sciences version 22. Bivariate and multivariate logistic regressions were done. An adjusted odds ratio at a *p* value < 0.05 with 95% confidence interval was taken to declare statistical significance.

**Result:**

A total of 644 students have participated with a response rate of 97.7%. The prevalence of risky sexual behavior among adolescents aged 15-19 years was found to be 17.2%. Factors like poor social support (AOR = 5.59, 95% CI: 2.71-11.53), living out of family (AOR = 1.93, 95% CI: 1.21-3.07), experiencing parental neglect (AOR = 1.87, 95% CI: 1.18-2.94), and drinking alcohol (AOR = 2.55, 95% CI: 1.55-4.20) were statistically associated with risky sexual behavior. *Conclusion and Recommendations*. The prevalence of risky sexual behavior was found to be alarming among adolescents of high school aged 15-19 years. This can significantly affect health quality in the community and the country at large. We recommend setting strategies that are against the determining factors of risky sexual behavior; the control of alcoholic beverages among adolescents aged 15-19 years must be enhanced, and awareness creation must be made regarding its unpleasant consequences.

## 1. Introduction

Adolescence is a critical age in which people need to undergo sexual development [1]. Many health risks and complications secondary to unprotected sexual activity among adolescents have been documented by the World Health Organization (WHO) [2, 3], for which interventions should be implemented promptly. Recent data indicated that 1.2 billion people in the world are adolescents aged 10-19 years [4].

Adolescents are slightly at an increased level of vulnerability for different health conditions including sexually transmitted diseases when compared to the adult population [5]. Risky sexual behavior is characterized by different hazardous behaviors such as premarital sex, multiple sexual partners, and unprotected sex. Such hazardous sexual behaviors are reported to end up with unpleasant health outcomes like HIV/AIDS, unwanted pregnancies, and unsafe abortions [6].

Health problems among adolescents including sexually transmitted infections linked with socioeconomic disadvantages seem to be increasing. Incidence and prevalence estimates indicated that one in four sexually active adolescent women has sexually transmitted infections such as chlamydia or human papillomavirus [7]. Compared with older adults, sexually active adolescents aged 15-19 years are at higher risk of acquiring sexually transmitted infections for the combination of behavioral, biological, and cultural reasons [8].

Unsafe sexual behavior and the associated exposure to infection is one of the major causes of preventable mortality in low-income countries (after childhood underweight and unsafe water) [9]. It is the major means of transmission for HIV/AIDS and human papillomavirus, with overall mortality more than one million people worldwide [10]. Among the means of transmission for HIV/AIDS in each state of America, the primary one is risky sexual behavior [11].

Pieces of literature have measured and reported the size of risky sexual behavior, and it ranges from 21.6 to 42.1% using different screening tools [12–15]. In one cross-sectional study conducted in the eastern part of Ethiopia among high school and preparatory students, they reported a 13.7% prevalence of risky sexual behavior [16].

Few studies have revealed its association with smoking, alcohol, and drug abuse, which are also considered risky behaviors or substance abuse. Shreds of evidence indicated that those adolescents who have experienced abuse by others and had antisocial behaviors have been found to have an increased chance of involving in risky sexual activities [17, 18].

Socioeconomic status, joblessness, sexually active friends, family instability, single-parent household, sibling sexual activity, and each characteristic (race, gender, age, and puberty status) have all been associated with adolescent risky sexual behavior [19].

Even though little is known about the size of risky sexual behavior, its size has been given little attention among adolescents particularly those aged 15-19 years. Understanding sexual behavior and its determinants among adolescents aged 15-19 years is crucial to come up with effective intervention. Therefore, this study investigated the magnitude of risky sexual behavior and its correlates among adolescents aged 15-19 years.

## 2. Materials and Methods

### 2.1. Study Design, Area, and Period

We conducted an institution-based cross-sectional study design among adolescents aged 15-19 years at governmental high schools in Aksum town, Tigray, Ethiopia. There are three governmental high schools in Aksum town named Kedamay-Minilik secondary school, Atse-Kaleb secondary school, and Aksum secondary school. A total of 2579 grade nine and 2241 grade ten students aged 15-19 years were attending their regular classes in the three high schools. Data were collected from January 1 to 30, 2019.

### 2.2. Sample Size Determination and Sampling Procedure

We calculated the sample size by using the single population proportion formula by taking the following assumptions: 1.96, *Z* (normal distribution), 95% confidence interval (CI) of certainty (*a* = 0.05), and a 5% marginal error. The proportion of risky sexual behavior was taken to be 51.3% from the previous study [20] and a 10% nonresponse rate. Therefore, based on the assumptions, the final sample size was taken to be 659.

A systematic random sampling technique was used to select a total of 659 adolescent participants aged 15-19 years during the study period. We considered all adolescent students aged 15-19 years in Aksum town in 2019 as a source population and all adolescent students aged 15-19 years during the data collection period as a study population. All students aged 15-19 years who already registered on the list were included, and those students aged 15-19 years who were critically sick during data collection and unable to communicate were excluded from the study.

### 2.3. Outcome Variable

The outcome variable for this study was risky sexual behavior. It was measured by a five-item screening tool that was prepared by adopting the World Health Organization Sexual and Reproductive Health (SRH) questionnaire for local-based scenarios. The screening tool was used before in our country, and reliability Cronbach's alpha was reported to be 0.92 [16]. The sexual risk behavior scale (SRBS) was used to assess the lifetime risky sexual activities of the adolescents. The items ask about the presence or absence of vaginal sexual intercourse, early sexual debut (before 14 years of age), having multiple sexual partners, having HIV testing, and inconsistent condom use in their sexual practices. Adolescents aged 15-19 years who have practiced in at least one were considered positive for risky sexual behavior [16].

### 2.4. Independent Variables

#### 2.4.1. Sociodemographic Factors

Sociodemographic variables included age, sex, religion, ethnicity, educational level, residence, number of children, the educational status of the mother, educational status of the father, and occupational status of fathers and mothers.

#### 2.4.2. Depression and Parental Neglect

Public health questionnaire 9 (PHQ-9) was used to assess depression which is a multipurpose instrument for screening, diagnosing, monitoring, and measuring the severity of depression. Those who scored >5 from the PHQ-9 scale were considered possibly positive for depression [21]. The adverse childhood experience questionnaire which is a 10-item screening tool was used to access parental neglect. The adverse childhood experience questionnaire includes questions that assess emotional abuse and neglect, physical abuse and neglect, educational and medical neglect, and sexual abuse [22].

#### 2.4.3. Social Support and Substance-Related Factors

The level of social support was assessed by the Oslo-3 social support scale. The scale divides the level of social support into three as poor social support (3-8), moderate social support (3-14), and strong social support (12-14) (reliability Cronbach′s *α* = 0.91) [23]. Substance use history (alcohol, khat, and cigarette) was assessed by yes/no questions for current use and lifetime use.

### 2.5. Data Collection Procedure

Data were collected as self-reported using a structured questionnaire. The study subjects were informed about the general information about the study objectives as well as the opportunities or benefits that this study could bring. Finally, the filled questionnaires were checked for consistency and completeness daily. To assure the data quality, high emphasis was given in designing data collection instruments. Structured and pretested questionnaires were used to collect information. The overall questionnaires were prepared in English, and it was translated to the local language “Tigrigna”; finally, we translated it back to English to check the consistency of the information to be collected. The training was given for data collectors and supervisors by the principal investigator on the methods of data collection for two days. The questionnaire was pretested one week before the actual data collection on 5% of the total same size that was not included in the main survey.

### 2.6. Data Processing and Analysis

The entire questionnaires were checked for completeness and entered into EpiData 4.1, and then, it was exported to SPSS 22 version statistical software for analysis. Descriptive statistics, bivariate analysis, and multivariate logistic regression were conducted. The sociodemographic characteristics of respondents were analyzed by descriptive statistics. Univariate analysis was used to see the association between risky sexual behavior and independent variables. Variables whose *p* value is <0.2 were entered to multivariate logistic regression to control confounding effect. The significance was declared at *p* value < 0.05. To determine the strength of association between dependent and independent variables, the adjusted odds ratio at a 95% confidence interval was used.

## 3. Result

### 3.1. Sociodemographic Characteristics

A total of 644 individuals aged 15-19 years participated in the study with a response rate of 97.7%. More than half 55% (354) of the study participants were female students. The majority of participants, 78.6% (506) of study participants, were Orthodox Christian followers by religion. Regarding educational levels of students, 53.2% (336) were grade nine students and the rest 47.8% (308) of them were grade ten students. One-third (67.1%) of the study participants were from urban in their residence (Table 1).

### 3.2. Social Support-Related Factor

**Figure 1:**
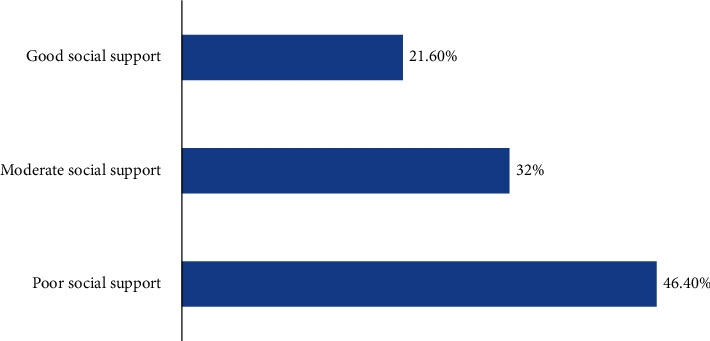
Distribution of social support-related factors among adolescents aged 15-19 years at high schools in Aksum town, Tigray, Ethiopia, 2019.

As measured by the three-item Oslo social support scale, 46.4% (299) of adolescents aged 15-19 years lay in the poor social support level followed by moderate social support which was 32% (206). The rest have been found to lay in a strong or good social support level (Figure 1).

### 3.3. Substance Use-Related Factors

**Figure 2:**
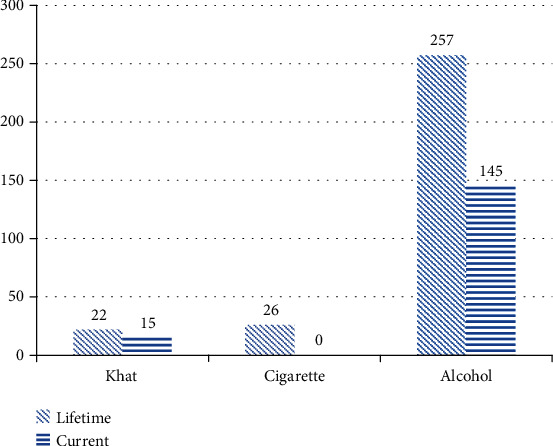
Distribution of substance use-related factors among adolescents aged 15-19 years at high schools in Aksum town, Tigray, Ethiopia, 2019.

Among participants of this study, most students used alcoholic beverages both in their lifetime and within the last three months of the study time (Figure 2).

### 3.4. Clinically Related Factors

Among the total participants of this study, 28.9% of them have been found to have possible depression as screened by PHQ-9. Parental treatments towards adolescents were also screened, and the result indicated that about 36.5% of the students reported that they are neglected by their parents, caregivers, or guardians during the study time.

### 3.5. Prevalence of Risky Sexual Behavior and Associated Factors

**Figure 3:**
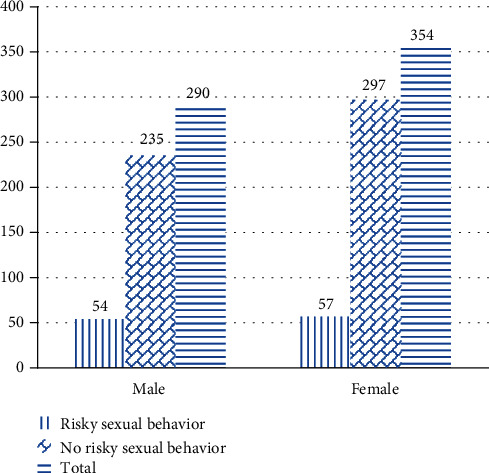
Distribution of prevalence of risky sexual behavior with sex of participants among adolescents aged 15-19 years at high schools in Aksum town, Tigray, Ethiopia, 2019.

According to our study, the overall prevalence of risky sexual behavior among adolescents aged 15-19 years at high schools in Aksum town was found to be 17.2% (95% CI; 14.3%, 20.2%). Among the total 354 female participants, risky sexual behavior is 16.1% (57/354) whereas it is found to be 18.6% (54/290) in males. A high degree of occurrence in the prevalence of risky sexual behavior has been observed among those who were following grade nine when compared to those who were following grade ten during the study period. On the contrary, almost the same degree of occurrence of risky sexual behavior has been observed between those who were from urban and rural (Figure 3).

Regarding factors associated with risky sexual behavior of study participants, bivariate logistic regression was done to identify candidate factors for multivariate logistic regression analysis. These factors have been selected at *p* value < 0.25. After controlling for confounding effect by multivariate logistic regression, factors like poor social support, living out of the family, experiencing parental neglect, and current alcohol use are associated with risky sexual behavior of study participants at *p* value < 0.05.

Those adolescent students aged 15-19 years who have poor social support were 5.59 times more likely to participate in risky sexual behavior as compared to those who have strong social support (AOR = 5.59, 95% CI: 2.71-11.53). Another variable that was found to be associated with risky sexual behavior was living without a family member. Students who were living out of their family members were 1.93 times more likely to participate in risky sexual behavior when compared to those who were living with their family (AOR = 1.93, 95% CI: 1.21-3.07).

Adolescent students aged 15-19 years who experienced parental neglect by their primary caregivers were also among those who were found to have a significant association with risky sexual behavior. The probability of participating in risky sexual behavior was found to be increased by 87% in adolescents aged 15-19 years who experienced parental neglect when compared to those adolescents aged 15-19 years who did not experience (AOR = 1.87, 95% CI: 1.18-2.94).

Those who were drinking alcoholic beverages were 2.55 times more likely to have risky sexual behavior than those who did not drink (AOR = 2.55, 95% CI: 1.55-4.20) (Table 2).

## 4. Discussion

### 4.1. Discussion on the Prevalence of Risky Sexual Behavior

Our study tried to add knowledge regarding the magnitude of risky sexual behavior and factors which may have an impact on the possibility of participating in such behavior among this special population.

The overall prevalence of risky sexual behavior among adolescent students aged 15-19 years in our study was 17.2%. Our study result is consistent with other studies conducted in Northwest Ethiopia among high school students and reported 19.8% [24], and 17.9% on a study done at Bodti, Ethiopia [25]. However, our study result regarding the prevalence of risky sexual behavior among adolescents aged 15-19 years is lower than that of the previous studies conducted in different parts of the world such as 51.3% in Mizan Tepi [20], Ethiopia; 43.1% in Addis Ababa [26]; 44.9% at Bahir Dar, Ethiopia [6]; 40.6% in another Bahir Dar, Ethiopia, study [27]; and 54% in the rural part of Cameroon [28]. This disparity might be due to the difference in study participants, in which adult construction works and college-level students were mainly considered in studies conducted at Bahir Dar, Ethiopia; preparatory students were also included in a study done at Mizan Tepi, Ethiopia; clinical patients were studied at Addis Ababa; and only female students were studied in a Cameroon study. Another possible reason for such difference might also be the cultural influence on adolescents and problems on their degree of openness to report their sexual practices even it was self-reporting.

On the other hand, the current study finding for risky sexual behavior among adolescents aged 15-19 years was higher than that of the study conducted among adolescents at Humera, Ethiopia, 13.7%. This disparity might be due to the difference in the study participants, in which preparatory school students were also included in a study at Benishangul Gumuz, Ethiopia [24]. This might also be due to the difference in sample size, in which only 422 students were included in the study conducted at Humera, Ethiopia [16].

### 4.2. Discussion on Factors Associated with Risky Sexual Behavior

Those adolescents aged 15-19 years who have poor social support were 5.59 times more likely to participate in risky sexual behavior when compared to those who have strong social support. This might be because the absence of supportive and caring relationships with families and other school communities leads adolescents to poor academic achievement. This poor academic failure is one of the major contributing factors in participation in risky health behavior [29]. This is supported by different studies [30–32].

Adolescents aged 15-19 years who were living without their family were 1.93 times more likely to have risky sexual behavior when compared to those who live with their families. This might be because adolescent age group close family supervision hinders them from participating in different risky behaviors particularly sexual activities. This might also be due to the reason that peer pressures get stronger to influence and make them participate in such risky behaviors when their close family member is not around [33]. This study was supported by different studies conducted earlier in different areas [16].

This study further explored that the risk of participating in risky sexual activities increased by 87% in those adolescents aged 15-19 years who experienced parental neglect by their primary caregiver when compared to adolescents aged 15-19 years who did not. This might be due to the increased emotional disturbances soon as they experience neglect which leads them to participate in risky behaviors as a defense mechanism [34, 35]. Different studies conducted earlier are alongside this study.

Moreover, this study declared that using alcoholic beverages has a significant influence on the fate of participation in risky sexual activities. Those adolescents aged 15-19 years who used alcohol within three months before the start of the study period were 2.55 times more likely to participate in risky sexual activities than those who did not use alcohol. This might be because almost all mental functions get lowered by the effects of alcohol. This does not give them the chance to comprehend things that put them at potential risk to engage in risky behaviors. This might also be because alcohol use has been linked to early sexual debut and increased likelihood of adolescents being sexually active while they are at school [36, 37]. This study has been supported by other studies [36, 37].

## 5. Conclusion and Recommendation

This study has declared that an alarming proportion of adolescent students aged 15-19 years have participated in risky sexual behavior. This can significantly affect the quality of health in the community and the country at large. Factors, like having poor social support, living out of the family, experiencing parental neglect, and using alcoholic beverages, are risk factors that increase the odds of risky sexual behavior among adolescent students aged 15-19 years.

An action shall be taken which are against the factors found to increase the odds of risky sexual behavior to protect the health of students aged 15-19 years while they are at school. We would like also to recommend that the control of alcoholic beverages among adolescents aged 15-19 years must be enhanced and awareness creation must be made regarding its unpleasant consequences, especially on these special populations.

## Figures and Tables

**Table 1:** Sociodemographic characteristics of adolescents aged 15-19 years at high schools in Aksum town, Tigray, Ethiopia, 2019 (*n* = 644).

Variables	Category	Frequency	Percentage
Sex	Male	290	45
	Female	354	55
Educational level	Grade 9	336	52.2
	Grade 10	308	47.8
Religion	Orthodox Christian	512	79.5
	Muslim	106	16.5
	Protestant Christian	26	4
Residence	Urban	432	67.1
	Rural	212	32.9
Fathers' educational status	No formal education	86	13.4
	1-4th grade	169	26.2
	5-8th grade	146	22.7
	9-12th grade	113	17.5
	Above 12th grade	130	20.2
Mothers' educational status	No formal education	180	28
	1-4th grade	143	22.2
	5-8th grade	128	19.9
	9-12th grade	121	18.8
	Above 12th grade	72	11.2
Fathers' occupation	Farmer	228	35.4
	Daily laborer	34	5.3
	Merchant	114	17.7
	Governmental/private employee	268	41.6
Mothers' occupation	Farmer	182	28.3
	Daily laborer	38	5.9
	Merchant	78	12.1
	Governmental/private employee	203	31.6
	Housewife	143	22.2
Family size	1-5	378	58.7
	>5	266	41.3

**Table 2:** Bivariate and multivariate logistic analysis of factors associated with risky sexual behavior among adolescents aged 15-19 years at high schools in Aksum town, Tigray, Ethiopia, 2019 (*n* = 644).

Variables	Category	Risky sexual behavior		COR (95% CI)	AOR (95% CI)
		Yes	No		
Educational level	Grade 9	65	271	1	1
	Grade 10	46	262	0.73 (0.49, 1.11)	0.72 (0.49, 1.13)
Social support	Poor	214	85	5.12 (2.57, 10.22)	5.59 (2.71, 11.53)^∗∗∗^
	Moderate	190	16	1.09 (0.48, 2.47)	1.27 (0.54, 2.97)
	Strong	129	10	1	1
Living with family member	Yes	63	381	1	1
	No	48	152	1.91 (1.26, 2.91)	1.93 (1.21, 3.07)^∗∗^
Depression	Yes	42	144	1.64 (1.07, 2.52)	1.21 (0.74, 1.98)
	No	69	389	1	1
Parental neglect	Yes	54	181	1.84 (1.22, 2.79)	1.87 (1.18, 2.94)^∗∗^
	No	57	352	1	1
Current alcohol use	Yes	41	104	2.42 (1.56, 3.76)	2.55 (1.55, 4.20)^∗∗∗^
	No	70	429	1	1
Lifetime cigarette smoking	Yes	12	14	4.94 (2.02, 10.01)	4.52 (0.92, 22.19)
	No	99	519	1	1
Current cigarette smoking	Yes	8	12	3.37 (1.35, 8.46)	0.29 (0.05, 1.83)
	No	103	521	1	1
Fathers' educational status	No formal education	15	71	1.75 (0.80, 3.84)	0.86 (0.36, 2.04)
	1-4th grade	27	142	1.58 (0.79, 3.14)	1.04 (0.49, 2.18)
	5-8th grade	26	120	1.80 (0.89, 3.61)	1.15 (0.54, 2.44)
	9-12th grade	29	84	2.86 (1.43, 5.74)	1.92 (0.89, 4.14)
	Above 12th grade	14	116	1	1

^∗∗^ *p* value < 0.01; ^∗∗∗^*p* value < 0.001.

## Data Availability

The source data included in the manuscript can be accessed from the corresponding author Mengesha Srahbzu upon request through email address of mengusew@gmail.com.
